# Supplementary material for: Thousands of Pristionchus pacificus orphan genes were integrated into developmental networks that respond to diverse environmental microbiota
Source: PLoS Genet. 2023 Jul 3;19(7):e1010832. doi: 10.1371/journal.pgen.1010832 (PMC10348561; doi:10.1371/journal.pgen.1010832)
Supplement: S1 Fig — The scatter plots show the normalized expression (TPM) for different pairs of samples. As lowly expressed genes tend to be more variable, we visualized the number of genes with at least two-fold expression difference across multiple mean expression levels. (PDF) [file pgen.1010832.s001.pdf]

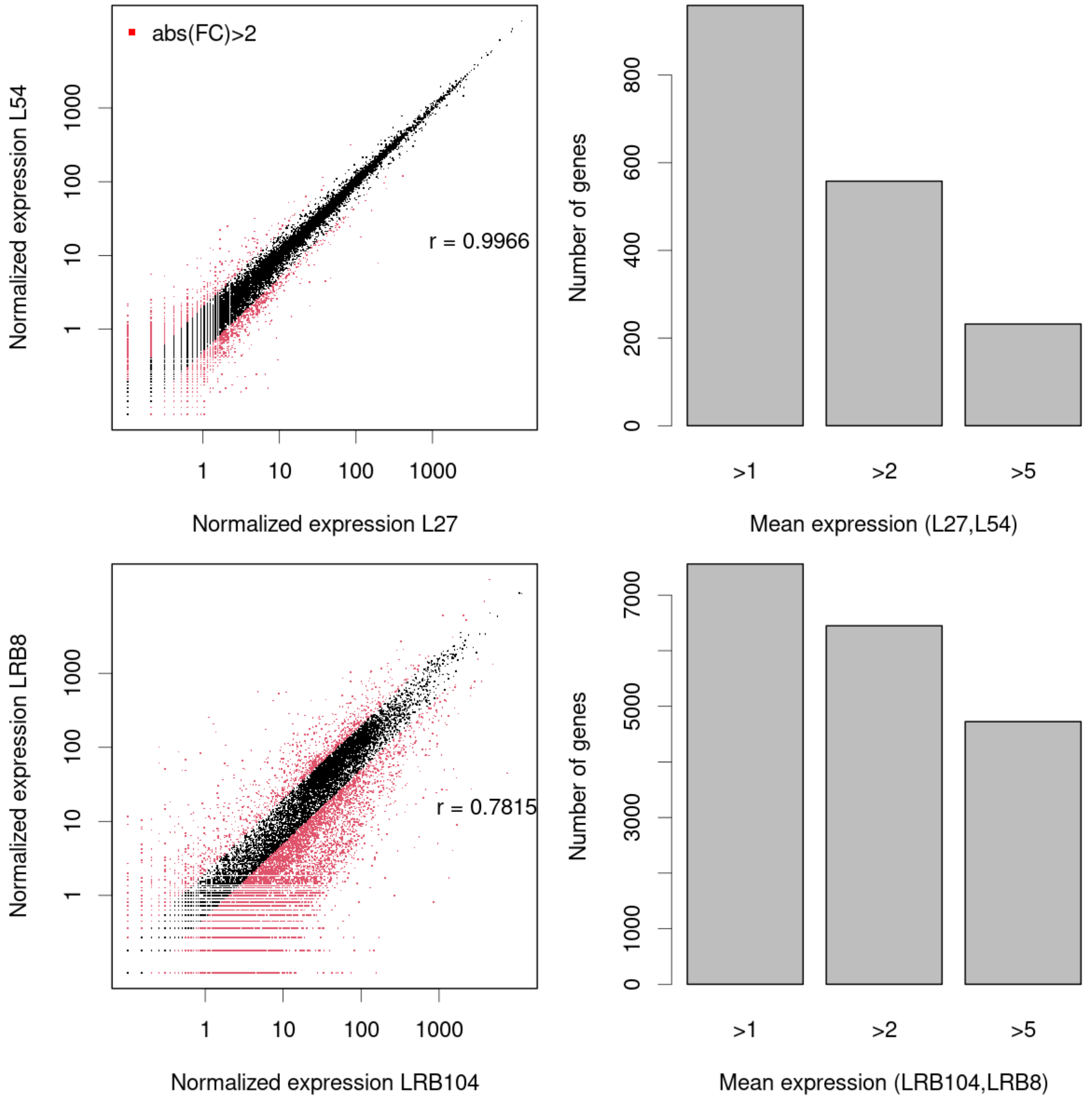

S1 Fig. Analysis of the most similar and dissimilar RNA-seq data sets. The scatter plots show the normalized expression (TPM) for different pairs of samples. As lowly expressed genes tend to be more variable, we visualized the number of genes with at least two-fold expression difference across multiple mean expression levels.
