# Supplementary material for: Thousands of Pristionchus pacificus orphan genes were integrated into developmental networks that respond to diverse environmental microbiota
Source: PLoS Genet. 2023 Jul 3;19(7):e1010832. doi: 10.1371/journal.pgen.1010832 (PMC10348561; doi:10.1371/journal.pgen.1010832)
Supplement: S2 Fig — We evaluated the total number of modules and singleton modules as a function of the inflation factor (I) and correlation coefficient (r). High r and I values generally increase the number of modules, whereas low r and I parameters generate fewer but larger modules. We decided to use r = 0.7 and I = 2 for the final analysis, because it gave a moderate number of modules with a relatively low number of singletons. (PDF) [file pgen.1010832.s002.pdf]

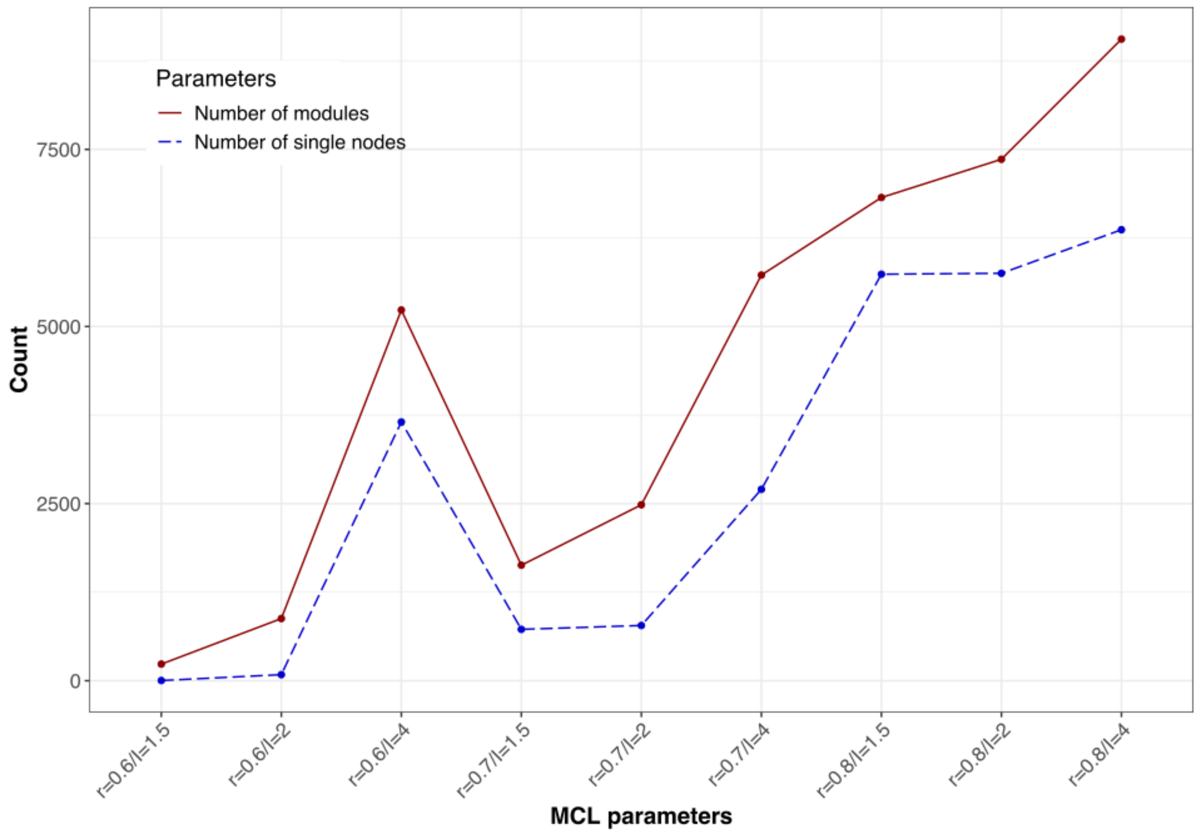

S2 Fig. Parameter combinations for MCL clustering. We evaluated the total number of modules and singleton modules as a function of the inflation factor (I) and correlation coefficient (r). High r and I values generally increase the number of modules, whereas low r and I parameters generate fewer but larger modules. We decided to use r=0.7 and I=2 for the final analysis, because it gave a moderate number of modules with a relatively low number of singletons.
