# Supplementary material for: Thousands of Pristionchus pacificus orphan genes were integrated into developmental networks that respond to diverse environmental microbiota
Source: PLoS Genet. 2023 Jul 3;19(7):e1010832. doi: 10.1371/journal.pgen.1010832 (PMC10348561; doi:10.1371/journal.pgen.1010832)
Supplement: S3 Fig — The distribution of developmental stages 56h after J2 synchronization is shown for P. pacificus worms on E. coli OP50 and two Wautersiella bacteria (5 biological replicates). Nematodes grow slower on both Wautersiella strains. The significance level was computed by a χ2 -test (mean P-value from all pairwise comparisons). (PDF) [file pgen.1010832.s003.pdf]

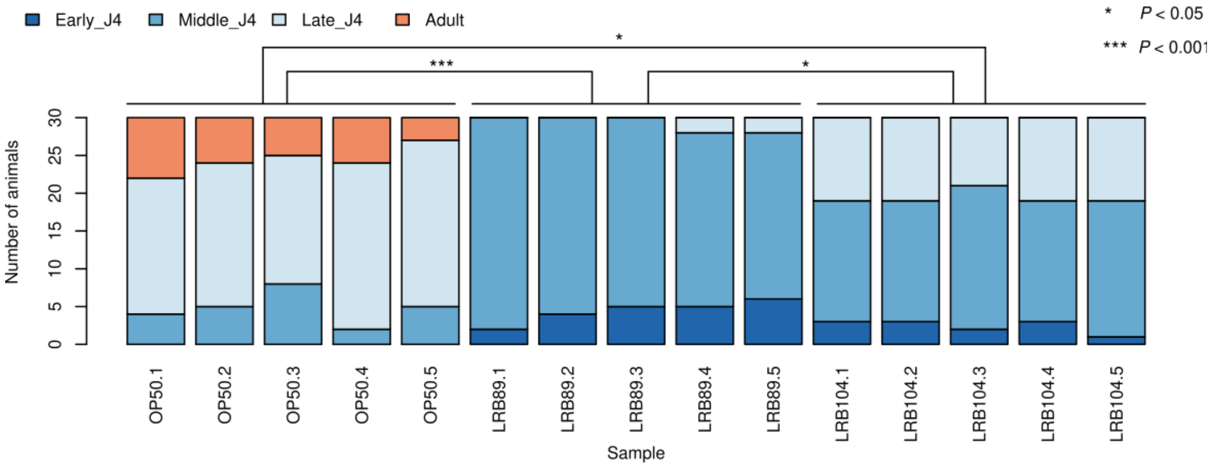

S3 Fig. Developmental timing on different bacteria. The distribution of developmental stages 56h after J2 synchronization is shown for *P. pacificus* worms on *E. coli* OP50 and two *Wautersiella* bacteria (5 biological replicates). Nematodes grow slower on both *Wautersiella* strains. The significance level was computed by a χ^2-test (mean *P*-value from all pairwise comparisons).
