# Supplementary material for: Thousands of Pristionchus pacificus orphan genes were integrated into developmental networks that respond to diverse environmental microbiota
Source: PLoS Genet. 2023 Jul 3;19(7):e1010832. doi: 10.1371/journal.pgen.1010832 (PMC10348561; doi:10.1371/journal.pgen.1010832)
Supplement: S4 Fig — The heatmap shows fraction genes from modules of the complete coexpression network (including LRB104) that overlap within a given module from the coexpression network without LRB104. For most modules there exists a 1–1 correspondence between both networks, indicating that the network structure is robust with regard to the Wautersiella LRB104 data set. (PDF) [file pgen.1010832.s004.pdf]

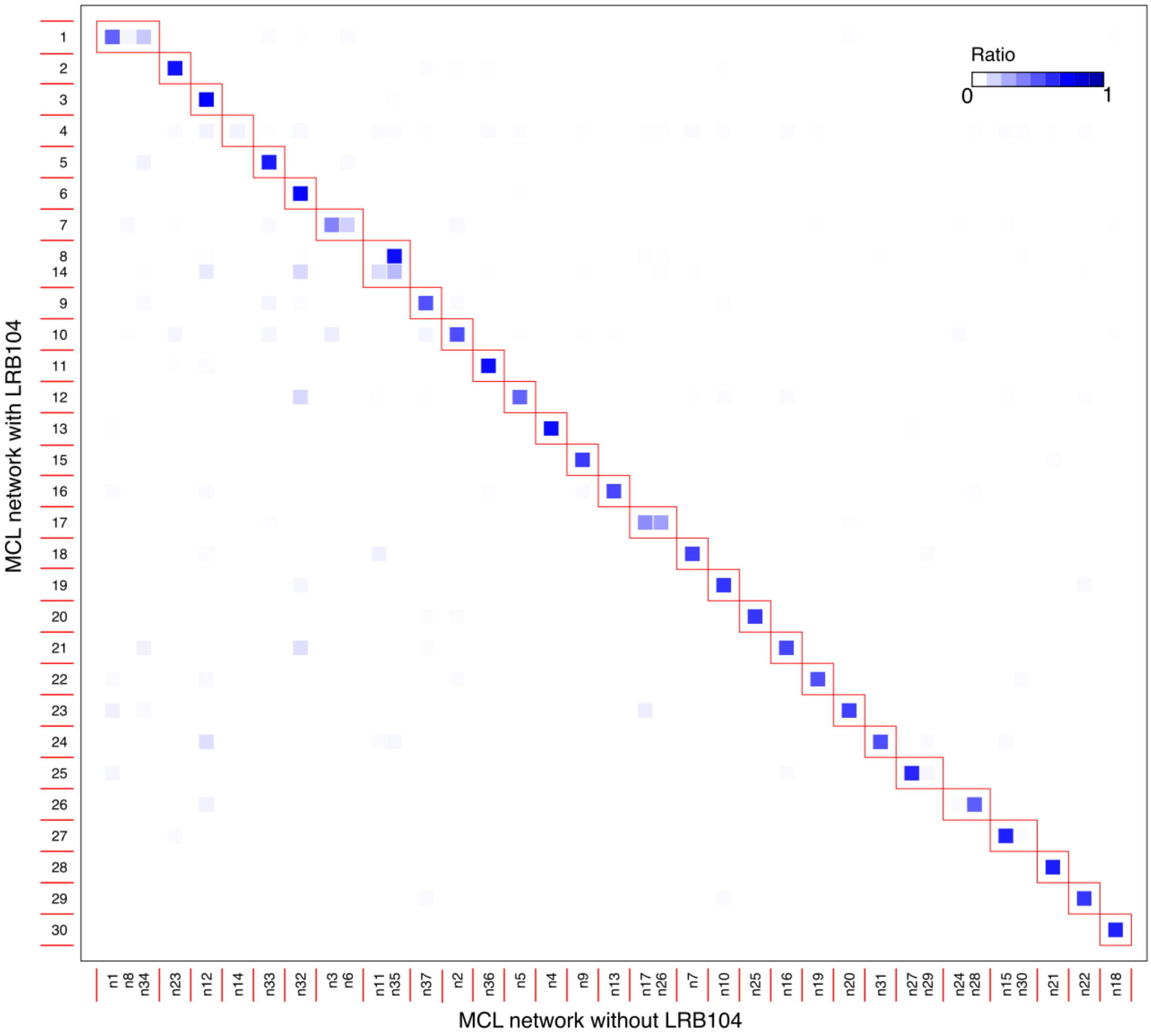

S4 Fig. Comparison of MCL networks constructed with or without LRB104. The heatmap shows fraction genes from modules of the complete coexpression network (including LRB104) that overlap within a given module from the coexpression network without LRB104. For most modules there exists a 1-1 correspondence between both networks, indicating that the network structure is robust with regard to the *Wautersiella* LRB104 data set.
