# Supplementary material for: Thousands of Pristionchus pacificus orphan genes were integrated into developmental networks that respond to diverse environmental microbiota
Source: PLoS Genet. 2023 Jul 3;19(7):e1010832. doi: 10.1371/journal.pgen.1010832 (PMC10348561; doi:10.1371/journal.pgen.1010832)
Supplement: S5 Fig — Taking the full coexpression network calculated from 24 RNA-seq samples as reference, we evaluated the classification of 10,000 randomly chosen gene pairs using subsampled RNA-seq data. For the full and the subsampled data set, a gene pair was either classified to be part of the same module or not. This allowed us to calculate the positive predictive value (PPV, panel A) and negative predictive value (NPV, panel B) for 10 randomly subsampled data sets of a fixed size. While the NPV is always close to 1, the PPV shows drastic differences between the full and subsampled data. This suggests that with fewer RNA-seq samples, additional gene pairs are assigned to the same coexpression module. However, with additional samples, such modules may be split into smaller components. Consistently, the number of genes in large modules (N>50) is much higher for smaller sample sizes (panel C). Panel D shows the number of diplogastrid-specific orphan genes in large modules. (PDF) [file pgen.1010832.s005.pdf]

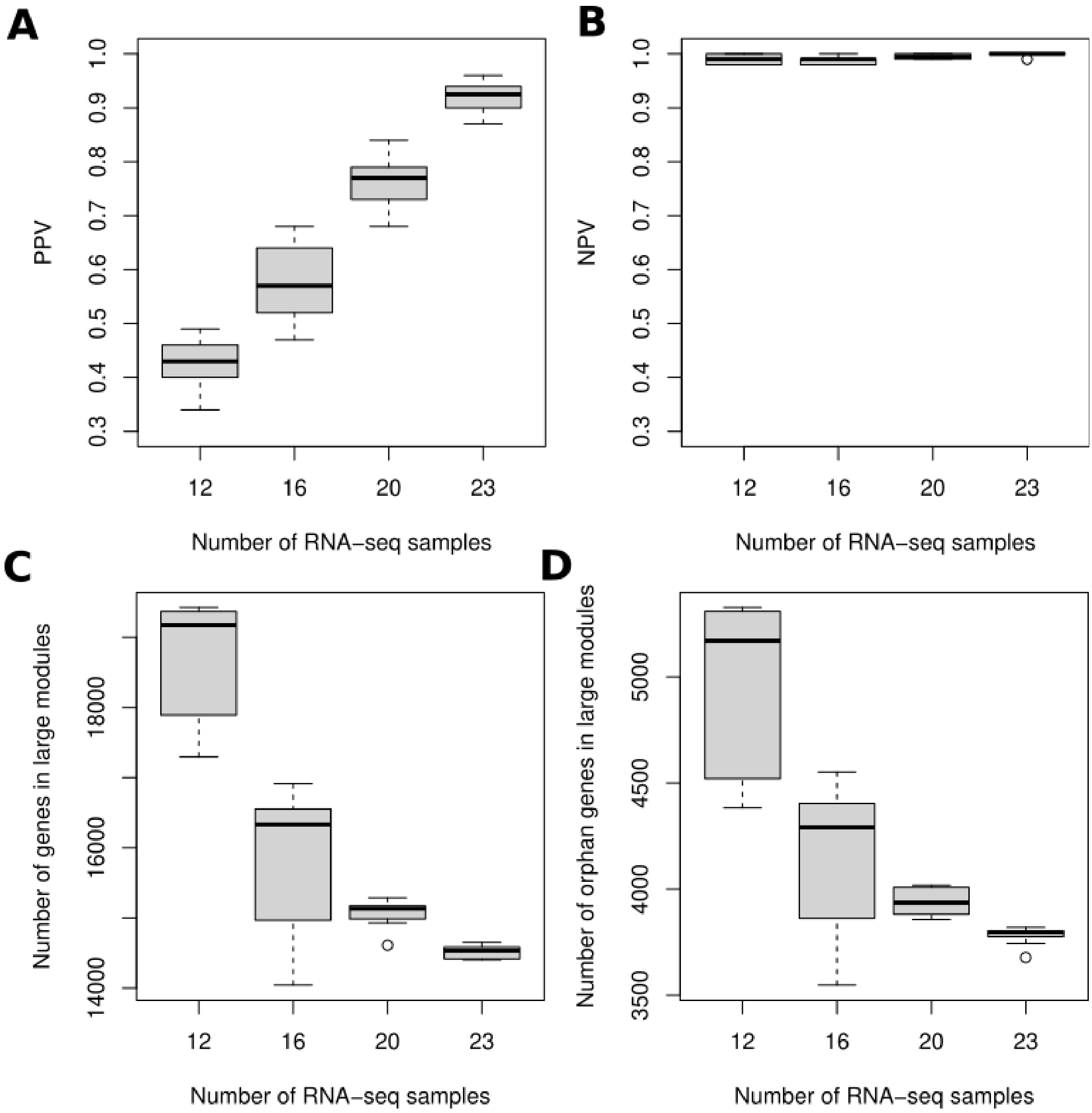

S5 Fig. Comparison of Coexpression networks on subsampled RNA-seq data. Taking the full coexpression network calculated from 24 RNA-seq samples as reference, we evaluated the classification of 10,000 randomly chosen gene pairs using subsampled RNA-seq data. For the full and the subsampled data set, a gene pair was either classified to be part of the same module or not. This allowed us to calculate the positive predictive value (PPV, panel A) and negative predictive value (NPV, panel B) for 10 randomly subsampled data sets of a fixed size. While the NPV is always close to 1, the PPV shows drastic differences between the full and subsampled data. This suggests that with fewer RNA-seq samples, additional gene pairs are assigned to the same coexpression module. However, with additional samples, such modules may be split into smaller components. Consistently, the number of genes in large modules (N>50) is much higher for smaller sample sizes (panel C). Panel D shows the number of diplogastrid-specific orphan genes in large modules.
