# Supplementary material for: Thousands of Pristionchus pacificus orphan genes were integrated into developmental networks that respond to diverse environmental microbiota
Source: PLoS Genet. 2023 Jul 3;19(7):e1010832. doi: 10.1371/journal.pgen.1010832 (PMC10348561; doi:10.1371/journal.pgen.1010832)
Supplement: S6 Fig — Significantly overrepresented motifs were identified for each module by the HOMER software. We arbitrarily selected motifs that occurred in at least 20% of promoters of a given module and visualized their distribution across all modules. Note that the complementary regulation by less frequent motifs and other regulatory mechanisms such as microRNAs are not considered here. Sequence logos for each motif are shown at the right and the labels to the left indicate the best motif match among known motifs. (PDF) [file pgen.1010832.s006.pdf]

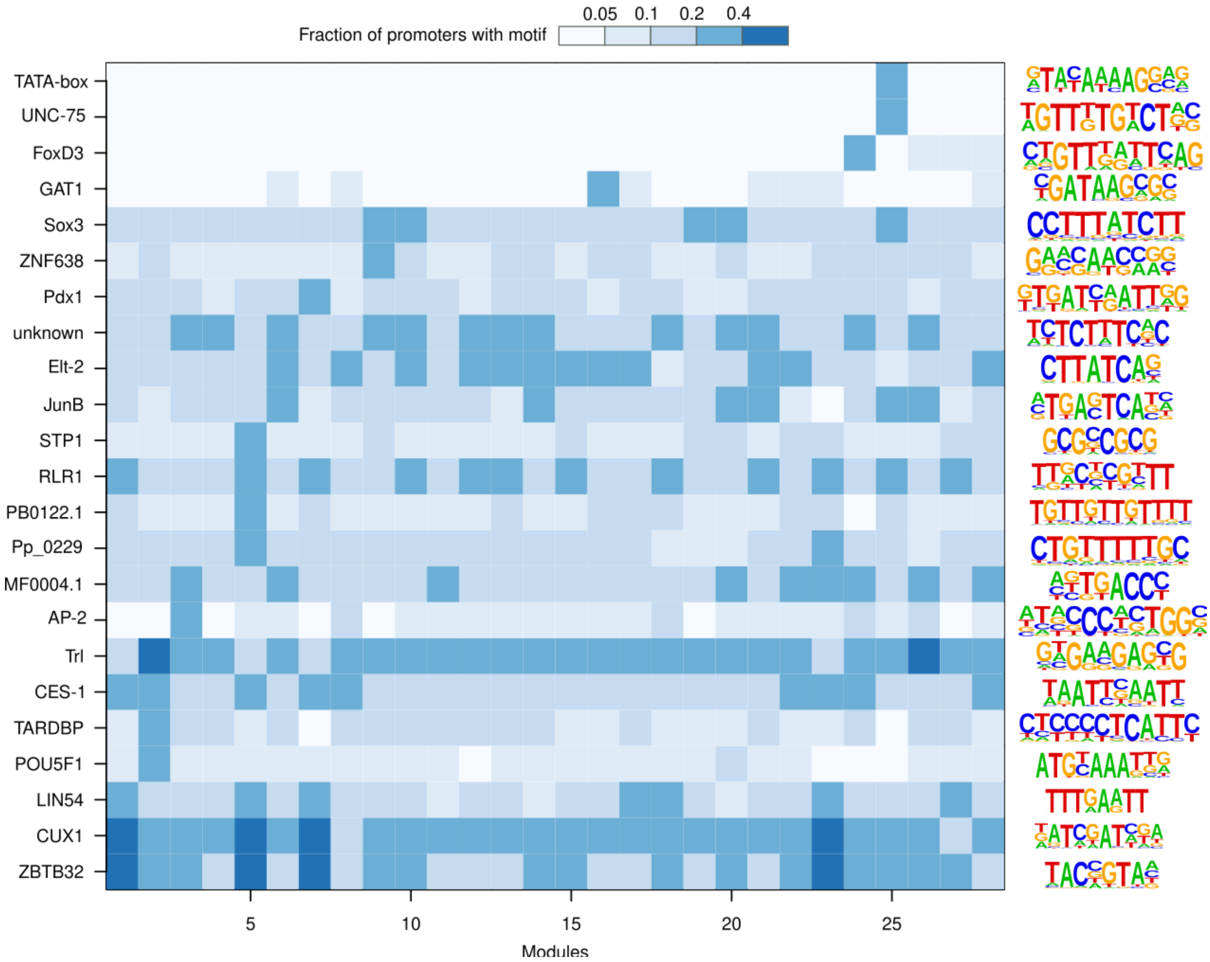

S6 Fig.. Regulatory architecture of coexpression modules. Significantly overrepresented motifs were identified for each module by the HOMER software. We arbitrarily selected motifs that occurred in at least 20% of promoters of a given module and visualized their distribution across all modules. Note that the complementary regulation by less frequent motifs and other regulatory mechanisms such as microRNAs are not considered here. Sequence logos for each motif are shown at the right and the labels to the left indicate the best motif match among known motifs.
