# Supplementary material for: Thousands of Pristionchus pacificus orphan genes were integrated into developmental networks that respond to diverse environmental microbiota
Source: PLoS Genet. 2023 Jul 3;19(7):e1010832. doi: 10.1371/journal.pgen.1010832 (PMC10348561; doi:10.1371/journal.pgen.1010832)
Supplement: S7 Fig — We simulated the integration of novel genes into existing networks by estimating ancestral module sizes based on the number of ancient genes (non-orphan genes) and then assigning an equivalent number of orphan genes to existing modules with equal probabilities (panel A and C) and with probabilities that were proportional to the module size (panel B and D). The scatterplots show the observed and simulated number of orphan genes per module. The barplots show the median enrichment of the observed relative to the simulated number of orphan genes (error bars indicate the minimal and maximal values from 100 simulations). (PDF) [file pgen.1010832.s007.pdf]

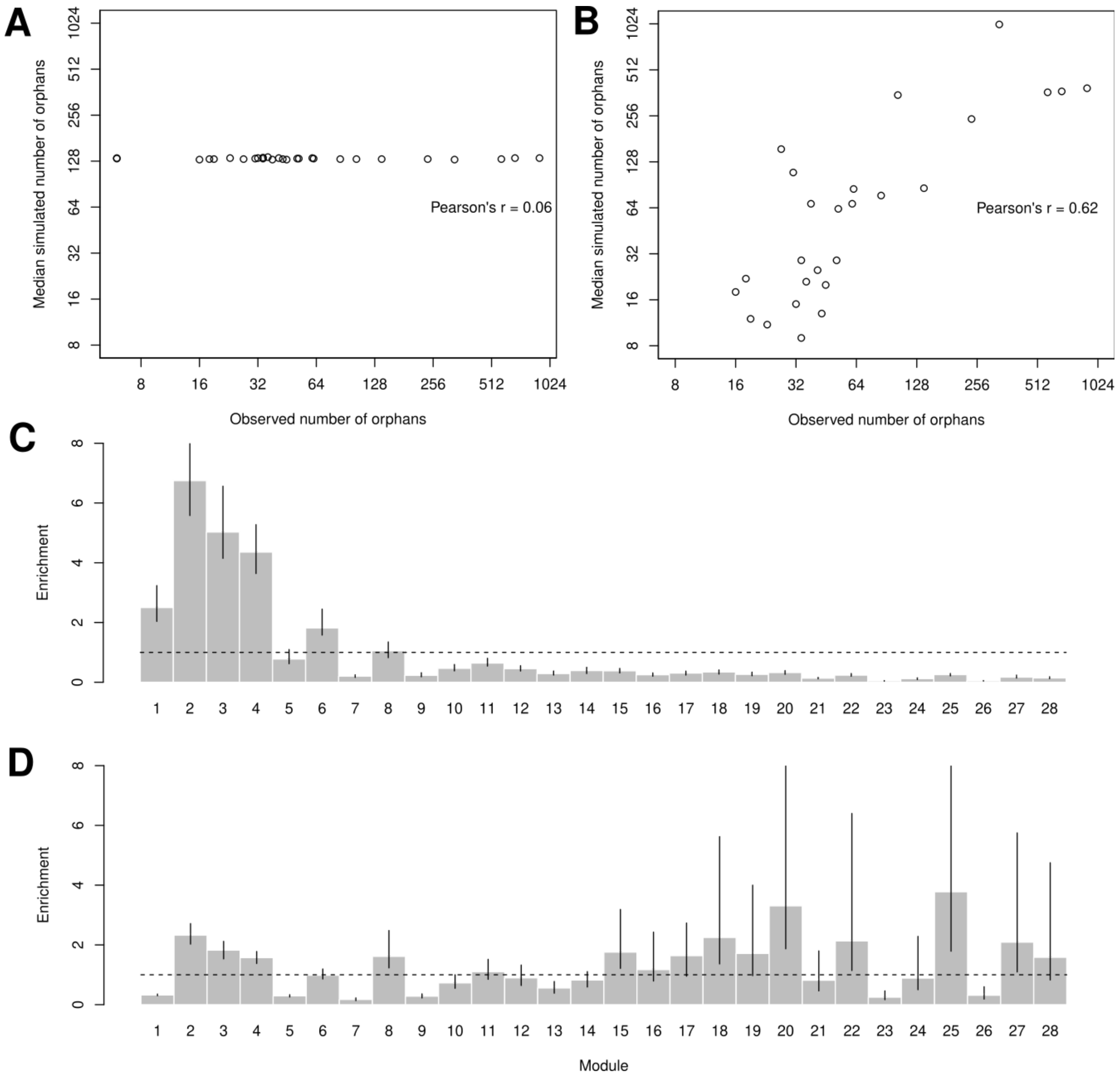

S7 Fig. Enrichment analysis for simulated network evolution. We simulated the integration of novel genes into existing networks by estimating ancestral module sizes based on the number of ancient genes (non-orphan genes) and then assigning an equivalent number of orphan genes to existing modules with equal probabilities (panel A and C) and with probabilities that were proportional to the module size (panel B and D). The scatterplots show the observed and simulated number of orphan genes per module. The barplots show the median enrichment of the observed relative to the simulated number of orphan genes (error bars indicate the minimal and maximal values from 100 simulations).
