# Supplementary material for: Thousands of Pristionchus pacificus orphan genes were integrated into developmental networks that respond to diverse environmental microbiota
Source: PLoS Genet. 2023 Jul 3;19(7):e1010832. doi: 10.1371/journal.pgen.1010832 (PMC10348561; doi:10.1371/journal.pgen.1010832)
Supplement: S8 Fig — Specific protein domains are strongly overrepresented in twelve coexpression modules. The left barplot shows the number of genes with a given protein domain for each module and the the right bar plot shows the negative logarithm of the FDR corrected P-value (Fisher’s exact test). The most significant association is between Motile sperm proteins (PF00635) and coexpression module 2. (PDF) [file pgen.1010832.s008.pdf]

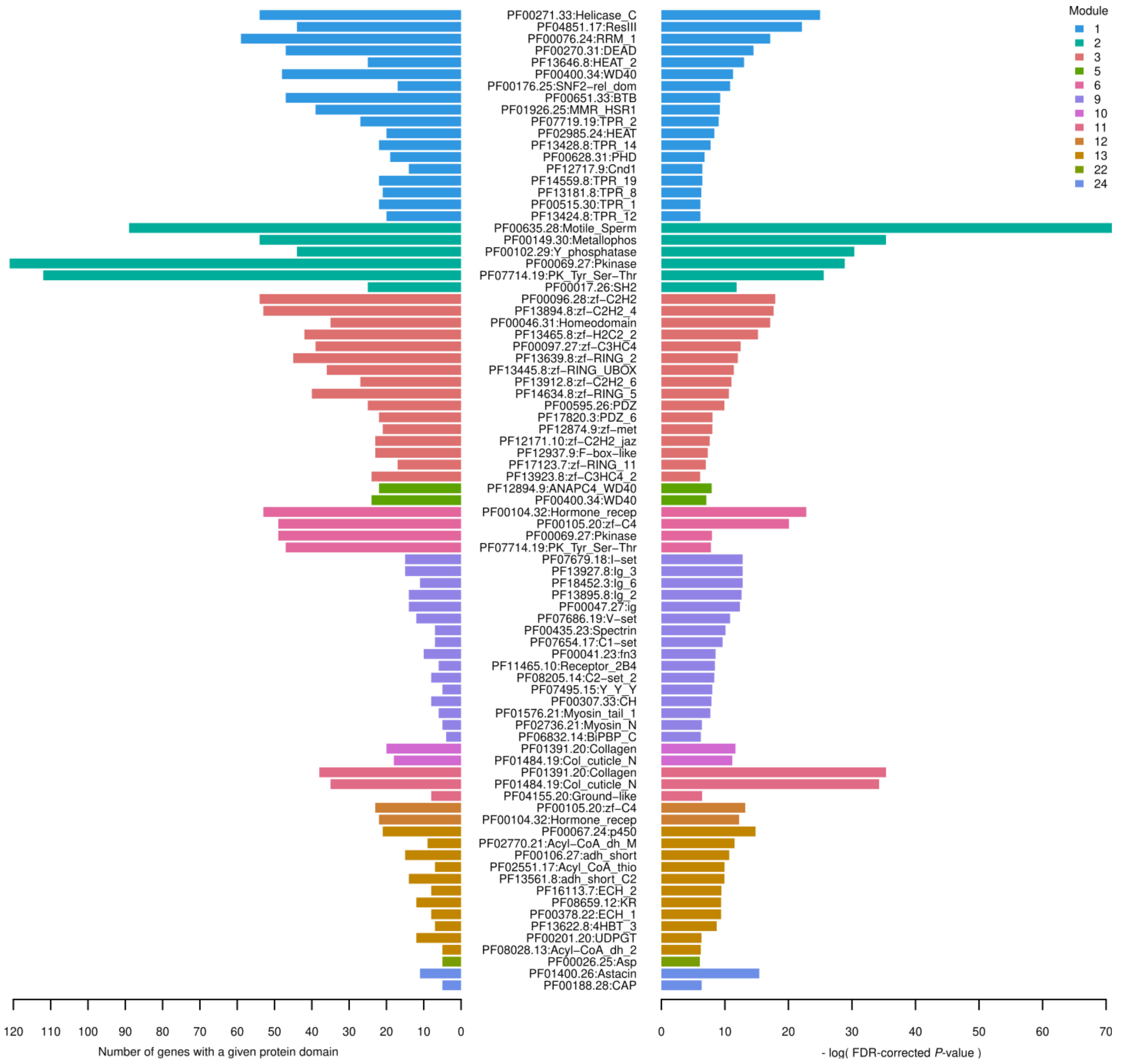

S8 Fig. Overrepresented protein domains in coexpression modules. Specific protein domains are strongly overrepresented in twelve coexpression modules. The left barplot shows the number of genes with a given protein domain for each module and the the right bar plot shows the negative logarithm of the FDR corrected P-value (Fisher's exact test). The most significant association is between Motile sperm proteins (PF00635) and coexpression module 2.
