# Supplementary figures and images for: Thousands of Pristionchus pacificus orphan genes were integrated into developmental networks that respond to diverse environmental microbiota
Source: PLoS Genet. 2023 Jul 3;19(7):e1010832. doi: 10.1371/journal.pgen.1010832 (PMC10348561; doi:10.1371/journal.pgen.1010832)

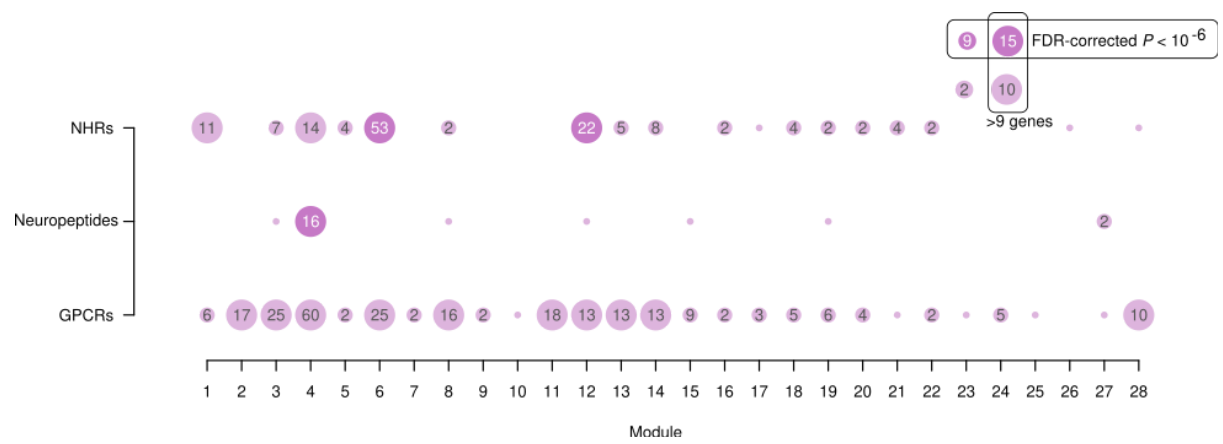

**S9 Fig. Distribution of GPCRs, NHRs, and neuropeptides across the coexpression modules.**

Supplement: S9 Fig — (PDF) [file pgen.1010832.s009.pdf]
