# Supplementary material for: Thousands of Pristionchus pacificus orphan genes were integrated into developmental networks that respond to diverse environmental microbiota
Source: PLoS Genet. 2023 Jul 3;19(7):e1010832. doi: 10.1371/journal.pgen.1010832 (PMC10348561; doi:10.1371/journal.pgen.1010832)
Supplement: S10 Fig — The heatmap shows the expression of genes that are shared between module 24 and the target genes of mouth form regulators and additional module 24 genes with 1–1 orthologs in C. elegans. The C. elegans ortholog of PPA25527 (D1044.3) is reported to be expressed in the gland cell and reporter lines of multiple candidate genes show expression in the gland of P. pacificus (Sieribriennikov et al. 2020) [70]. (PDF) [file pgen.1010832.s010.pdf]

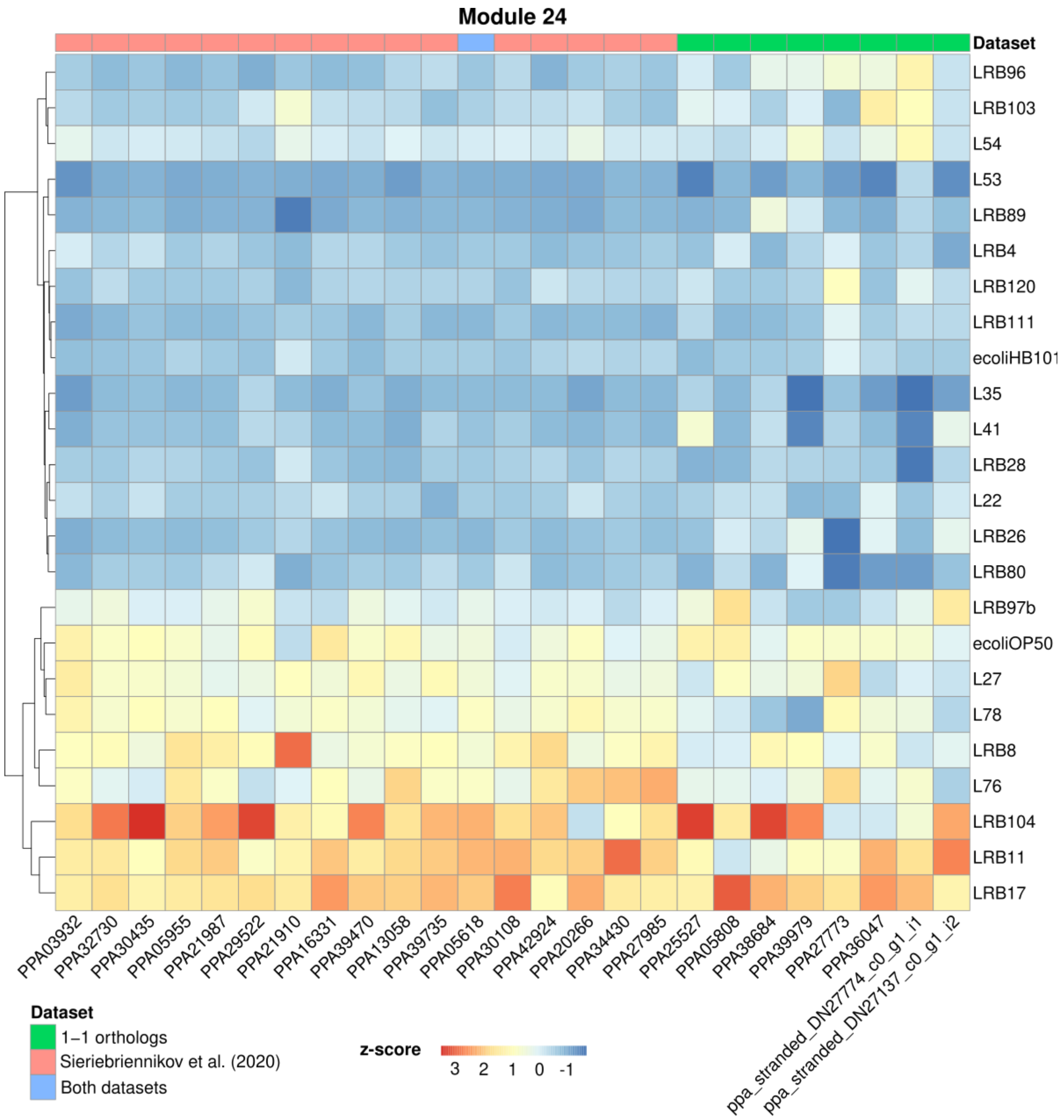

S10 Fig. Expression of selected module 24 genes. The heatmap shows the expression of genes that are shared between module 24 and the target genes of mouth form regulators and additional module 24 genes with 1-1 orthologs in *C. elegans*. The *C. elegans* ortholog of PPA25527 (D1044.3) is reported to be expressed in the gland cell and reporter lines of multiple candidate genes show expression in the gland of *P. pacificus* (Sieribriennikov et al. 2020) [1].

References

1. Sieriebriennikov B, Sun S, Lightfoot JW, Witte H, Moreno E, et al. Conserved nuclear hormone receptors controlling a novel plastic trait target fast-evolving genes expressed in a single cell. PLoS Genet. 2020;16: e1008687.
