# Supplementary material for: Thousands of Pristionchus pacificus orphan genes were integrated into developmental networks that respond to diverse environmental microbiota
Source: PLoS Genet. 2023 Jul 3;19(7):e1010832. doi: 10.1371/journal.pgen.1010832 (PMC10348561; doi:10.1371/journal.pgen.1010832)
Supplement: Attachment — Submitted filename: Response environmentally responsive orphans-1.pdf [file pgen.1010832.s017.pdf]

Reviewer #1:

Comment 1: *I appreciate the extra explanations and supporting information provided, which I believe has clarified key points and improved the manuscript. The revised version has now addressed all comments raised, although the limitation of dealing with single biological replicates remains.*
While the authors have reported and discussed this limitation, it may be worth reiterating it in the abstract to not mislead (careless) readers:
" Specifically, we performed RNA-seq experiments of P. pacificus worms grown on monoxenic cultures of 24 different bacteria" could be rephrased as follows: "Specifically, we analysed 24 RNA-seq samples from adult P. pacificus worms raised on 24 different monoxenic bacterial cultures".

Response: This was changed as proposed by the reviewer.

Comment 2: *I also have a couple of additional points that I would like to be addressed before publication: It is customary to provide a general method section that describes the way worms and bacteria are routinely maintained.*

Response: We have restructured the corresponding Methods according to these conventions.

Comment 3: *There are several instances of abbreviations used in Tables and Figures that are not spelled out in the legends. While some specialist may know exactly what they mean, the broader readership of PLoS Genetics would not.*

Response: Thanks for noticing this. We have now spelled out these abbreviations.

Comment 4: *Finally, there are issues with the way the pdf file comes out, so it would be worth ascertaining that the resolution of the figures is as intended before publishing it.*
I trust that all transcriptomics datasets (raw reads) will be made available for others to study.

Response: The reduced figure quality issue arose during the PDF conversion. The quality of the original tif files is fine. We confirm that all the raw reads are available on the European Nucleotide Archive (study accession: PRJEB60166).

Reviewer #2:

Comment: *The revisions made in the new version address my earlier concerns. Two small comments: 1 .It would be less confusing in Fig. 3 to distinguish the colors for modules from those for developmental stages.*

Response: We changed the color code for the developmental stages.

Comment: *2. In the abstract, line 50: "higher" than what? Could the author mean just "high"? or, "higher than expected by chance"?*

Response: Thanks, we have changed the wording to "considerably high".
